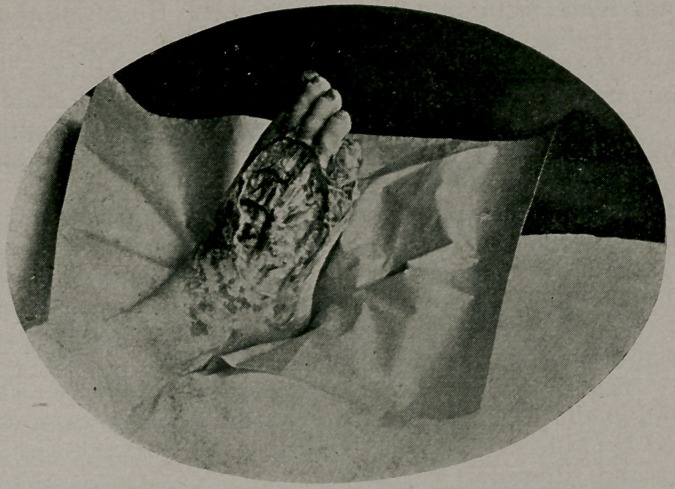# Raynaud’s Disease

**Published:** 1915-11

**Authors:** 


**Raynaud's Disease.** Harry Werner of Thomas, W. Va., Med. Summary, October, reports a case in a man aged 22. The patient was raised on a farm, there was no history of injury, urine negative, Wasserman negative, and the only etiologic factor discoverable was the strain and worry of a "business course"—which seems ludicrously insufficient. Healing was slow, the best results being ascribed to "echafolta" which we are unable to find indexed. The cut is loaned by the courtesy of the Editor.

## Figures and Tables

**Figure f1:**